# Peptides from Animal Venoms: A Promising Frontier in Diabetes Therapy via Multi-Target Mechanisms

**DOI:** 10.3390/ph18101438

**Published:** 2025-09-25

**Authors:** José Otávio Carvalho Sena de Almeida, Simón Gabriel Comerma-Steffensen, José Roberto de Souza de Almeida Leite, Ulf Simonsen, Daniel Dias Rufino Arcanjo

**Affiliations:** 1 LAFMOL–Laboratory of Functional and Molecular Studies in Physiopharmacology, Department of Biophysics and Physiology, Federal University of Piaui, Teresina 64049-550, Brazil; otavios.almeida@hotmail.com; 2 Graduate School of Pharmacology–PPGFarm, Medicinal Plants Research Center, Federal University of Piaui, Ministro Petrônio Portella Campus, Teresina 64049-550, Brazil; 3 Pulmonary and Cardiovascular Pharmacology, Department of Biomedicine, Aarhus University, 8000 Aarhus, Denmark; simoncomerma@biomed.au.dk (S.G.C.-S.); us@biomed.au.dk (U.S.); 4 Animal Physiology, Department of Biomedical Sciences, Faculty of Veterinary, Central University of Venezuela, Maracay 2105, Venezuela; 5 Research Group in Morphology and Applied Immunology, NuPMIA, Faculty of Medicine, University of Brasilia, Brasilia 70910-900, Brazil; jrleite@unb.br

**Keywords:** metabolic syndrome, therapeutic action, insulinotropic agents, insulin secretagogues, insulin mimetics, BLAST methodology

## Abstract

**Background/Objectives:** Bioactive peptides derived from animal venoms, toxins, and secretions demonstrate considerable pharmacological potential for use in the management of diabetes mellitus—a highly prevalent metabolic disorder of substantial global health significance. This integrative review systematically evaluated the current evidence regarding the pharmacological mechanisms underlying the antidiabetic properties of these bioactive peptides. **Methods:** This study was guided by the research question “What are the mechanisms of action of peptides derived from animal venoms in modulating parameters associated with diabetes?” developed using the PECo framework. A comprehensive literature search was executed across Scopus, PubMed, and Web of Science, focusing on studies from the last five years. Out of 190 identified articles, 17 satisfied the inclusion criteria. **Results:** Twenty-eight distinct peptides were characterized, exhibiting structural diversity with 7–115 amino acid residues and molecular weights of 900–13,000 Da. These compounds were sourced from venomous taxa including sea anemones, marine snails, spiders, centipedes, scorpions, and snakes. Their antidiabetic mechanisms encompassed glucagon-like peptide-1 (GLP-1) receptor agonism, insulin receptor activation, potassium channel inhibition, glucose transporter type 4 (GLUT4) upregulation, and α-amylase inhibition. Sequence analyses revealed substantial homology among peptides with analogous mechanisms—notably Con-Ins and ILP-Ap04, plus SpTx1 and SsTx-4—suggesting that structural determinants underlie their functional characteristics. Toxicological evaluations of nine peptides demonstrated low-toxicity profiles despite originating from toxic venom, crucial for therapeutic development. **Conclusions:** These peptides exhibited exceptional pharmacological potency with effective doses in nanogram-to-nanomole per kilogram ranges. Collectively, our findings underscore the therapeutic potential of venom-derived peptides as innovative candidates for use in diabetes management.

## 1. Introduction

Diabetes is classified as a metabolic disorder characterized by elevated serum glucose levels, leading to hyperglycemia. This condition is associated with disruptions in the metabolism of carbohydrates, lipids, and proteins and increased resistance to insulin action [1]. Diabetes is typically categorized into four types, with Type 1 and Type 2 being the most relevant to this discussion. Type 1 diabetes is primarily autoimmune in origin and involves the progressive destruction of pancreatic beta cells (β-PCs). This destruction results in the cessation of endogenous insulin production, making individuals reliant on external insulin therapy [2]. In contrast, Type 2 diabetes is characterized by chronic insulin resistance, which is often influenced by lifestyle factors such as diet and physical activity. This resistance leads to persistent hyperglycemia and other metabolic disturbances typical of the disease [3].

In 2022, the global prevalence of diabetes reached an estimated 828 million cases, with approximately 20 million new diagnoses annually. This rapid growth has led some researchers to classify diabetes as a global pandemic [2,4]. Contemporary therapeutic strategies for diabetes management encompass several pharmacological approaches: enhancement of insulin secretion using sulfonylureas and glucagon-like peptide-1 (GLP-1) receptor agonists, regulation of hepatic gluconeogenesis mediated by biguanides, and the use of sodium–glucose cotransporter-2 (SGLT-2) inhibitors, which function primarily to reduce the glycemic levels and thereby prevent or attenuate the late-stage complications associated with this metabolic disorder [5,6,7]. However, given the escalating number of individuals affected by this condition, there is growing concern that the existing therapeutic options may not be sufficient to meet the increasing demand [8]. Consequently, the development of novel pharmacological treatments that are more effective and potent, safer, and associated with fewer adverse effects is of critical importance [9]. Among the potential therapeutic agents, bioactive peptides derived from animal venoms, poisons, and secretions have shown immense promise [10]. These peptides exhibit unique pharmacological properties, including high specificity and potency, making them valuable candidates for addressing the challenges posed by diabetes treatment [11].

These bioactive substances have been extensively studied in the scientific literature due to their wide range of pharmacological activities, including their applications in pain management and antidiabetic treatments, antiparasitic effects, antimicrobial action, anti-inflammatory properties, applications in anticancer therapies, and antihypertensive effects [12,13,14,15]. Notably, some venom-derived peptides have already been successfully developed into marketable drugs. For example, Exenatide, a peptide drug of animal origin, is successfully used for the treatment of diabetes [16].

The body of research on the antidiabetic properties of peptides derived from animal sources is vast and spans several decades. It encompasses clinical studies and in vivo, in vitro, and in silico investigations. The diversity and volume of publications in this field underscore the need for a comprehensive synthesis of the recent findings. Therefore, this study aims to produce an integrative literature review identifying peptides of animal origin with demonstrated antidiabetic activity. Additionally, it seeks to elucidate their biological sources, the mechanisms through which they exert their pharmacological effects, and other key information necessary for a profound understanding of this topic.

## 2. Methodology

The central question guiding this integrative review was “What are the mechanisms of action of peptides derived from animal venoms in regulating parameters associated with the diabetic condition?” This question was structured using the PECo acronym (P: Problem; E: Exposure; Co: Context), as outlined in Table 1 [17].

The literature search used the Scopus, PubMed, and Web of Science databases, focusing on publications from the last five years (2020–2024); the cut-off date was set as December 20th 2024. The search strategy employed descriptors such as “venom”, “antidiabetic”, “diabetes mellitus”, “diabetes”, and “peptide”, which were combined using Boolean operators (AND/OR) to refine the results, as detailed in Table 2. This methodological approach ensured comprehensive coverage of recent studies relevant to the topic.

**Figure 1:**
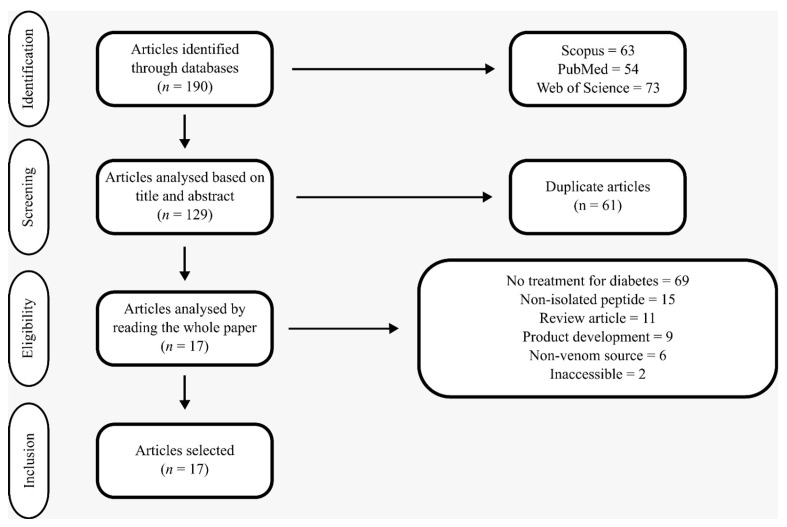
Modified PRISMA flowchart for articles selection.

After selecting and identifying articles from the databases, duplicate entries were removed. Subsequently, an eligibility analysis was conducted by reviewing the titles and abstracts of the remaining articles. At this stage, studies were excluded based on the following criteria: those not related to diabetes treatment; those not addressing the activity of isolated peptides of animal origin in in silico or in vitro studies; review articles; studies focused on the development of pharmaceutical products or formulations; studies not involving venoms, toxins, or animal secretions; and those that were inaccessible due to broken access links. The remaining articles were then read in full (Figure 1). The primary objective of this search was to identify studies published in English that evaluated isolated peptides derived from animal venoms, focusing on their potential mechanisms of action in the direct treatment of diabetes.

## 3. Results

Among the 17 articles analyzed, all the clinical studies focused on evaluating the drug Exenatide. Three of these studies investigated its effects on populations with diabetes to compare the treatment outcomes with those of a placebo [18,19,20]. Another clinical study explored potential pharmacogenomic effects associated with GLP-1 receptor activation by comparing Exenatide with a placebo in an Amish population in Pennsylvania [21]. Although other peptide-based drugs derived from animal venoms, such as Ziconotide, Lixisenatide, and Bivalirudin, are available on the market, no studies involving their use in diabetes treatment were identified [22].

The non-clinical studies utilized peptides obtained through various methods, including commercial synthesis, extraction from crude venom or secretions and purification, recombinant synthesis, solid-phase synthesis, and automated microwave synthesis with selective disulfide bridge formation. The in silico analyses employed homology-based approaches. The animal sources of these peptides were diverse and included sea anemones, marine snails, spiders, centipedes, scorpions, and snakes. A phylogenetic distribution could be established as follows: eight peptides were derived from the class Gastropoda, seven from Arachnida, six from Reptilia, five from Chilopoda, and two from Hexacorallia. The mechanisms underlying their antidiabetic activity were equally varied and included GLP-1 receptor agonism, insulin receptor agonism, potassium channel inhibition, increased expression of glucose transporter type 4 (GLUT4), and alpha-amylase inhibition.

Regarding the methodologies, the in vivo studies predominantly used mice as experimental models. The in vitro analyses frequently involved the BRIN-BD11, NIT-1, and INS-1 cell lines or assessed the enzymatic activity of alpha-amylases. The in silico studies focused on molecular docking analyses targeting key proteins relevant to diabetes treatment, such as the glucagon-like peptide-1 receptor (GLP-1R) and the insulin receptor (IR). Table 3 provides a detailed summary of the findings from each evaluated article.

A brief comparative analysis of peptides with similar mechanisms of action revealed a certain degree of homology in their amino acid sequences. Using the Basic Local Alignment Search Tool (BLAST) provided by the National Library of Medicine [36,37,38], it was observed that the peptides Con-Ins and ILP-Ap04, as well as SpTx1 and SsTx-4, exhibited an E-value greater than 1e–6 and 45% sequence identity. Notably, peptides exhibiting pronounced structural similarity originated from animal sources that shared a common phylogenetic classification at the class level. These findings indicate that these peptides’ activity on their respective targets has a degree of structural and functional dependence on their sequence composition (Table 4).

## 4. Discussion

### 4.1. Pharmacological Mechanisms in the Clinical Treatment of Diabetes

To comprehensively understand the mechanisms involved in diabetes treatment, it is essential to consider the physiological processes that regulate glucose metabolism within the body. This requires an understanding of the molecules responsible for this regulation, their mechanisms of action, their release into circulation, and the primary cell types involved in their response.

Before glucose enters the bloodstream, it must undergo digestion and absorption in the gastrointestinal tract from dietary starches. This process begins in the oral cavity and continues in the small intestine, where alpha-amylase enzymes break down polysaccharides into smaller subunits, ultimately yielding glucose. Glucose is then absorbed in the gastrointestinal tract through specific transporters [39]. Additionally, glucose is filtered in the kidneys and reabsorbed via sodium–glucose cotransporters type 2 (SGLT2) in the glomeruli. However, under diabetic conditions, this reabsorption becomes detrimental, making SGLT2 inhibitors a valuable therapeutic option [7].

β-PCs are highly sensitive to the glucose concentrations in the body. Glucose enters these cells and undergoes phosphorylation, which prevents it from exiting and directs it into the Krebs cycle. This process increases the intracellular adenosine triphosphate (ATP) levels, inhibiting the ATP-dependent potassium channels (K_ATP_). Blocking K_ATP_ channels facilitates membrane depolarization, triggers the opening of voltage-dependent calcium channels, and prolongs the depolarization period. These events initiate a cascade of responses that culminate in the fusion of insulin-containing vesicles with the cell membrane, releasing insulin into the bloodstream [40,41].

Glucagon-like peptide 1 receptor agonists (GLP-1RAs) act in this process by activating the Gs protein signal transduction pathway. This activation stimulates adenylate cyclase (AC), increasing the intracellular cyclic adenosine monophosphate (cAMP) levels and activating protein kinase A (PKA). PKA, in turn, facilitates membrane depolarization by opening sodium and calcium channels, thereby promoting insulin secretion [5]. GLP-1RAs are particularly effective in treating Type 2 diabetes mellitus (T2DM) during its early stages, as they enhance insulin secretion. However, their efficacy is limited in Type 1 diabetes mellitus (T1DM) and advanced-stage T2DM, where the β-PC functionality is significantly impaired unless some restoration is achieved [42,43,44]. Similarly, sulfonylureas act on β-PCs to stimulate insulin secretion. These drugs target K_ATP_ channels by binding to the sulfonylurea receptor subunits (SUR1s), thereby inhibiting channel opening. This inhibition prolongs the duration of cell depolarization, further facilitating insulin release [45,46].

Physiologically, hormones such as insulin, glucagon, and glucagon-like peptide 1 (GLP-1) play critical roles in regulating metabolism, necessitating precise control of their concentrations within the body. From a therapeutic perspective, dipeptidyl peptidase 4 (DPP- 4), an enzyme responsible for degrading GLP-1, is a significant target in diabetes treatment. Inhibiting DPP-4 increases the serum GLP-1 levels, allowing GLP-1 to maintain its insulinotropic activity and regulate the blood glucose effectively [47].

When insulin is present in the bloodstream, it acts on various cell types, including hepatocytes, adipocytes, and skeletal muscle cells. Insulin activates IRs, which are tyrosine kinases, initiating signal transduction through the phosphatidylinositol 3-kinase (PIK3) and protein kinase B (Akt) pathways. This signaling cascade facilitates the fusion of vesicles containing GLUT4 with the cell membrane [48,49]. GLUT4 on the membrane significantly enhances the uptake of glucose from the circulation, and glucose is subsequently utilized in anabolic processes such as triglyceride and glycogen synthesis [50,51]. Thiazolidinediones are a class of drugs that promote anabolism by targeting adipocytes. They interact with peroxisome proliferator-activated receptor gamma (PPARγ), inducing the expression of genes responsible for triglyceride synthesis and adipocyte proliferation. This mechanism contributes to improved blood glucose regulation [52].

**Figure 2:**
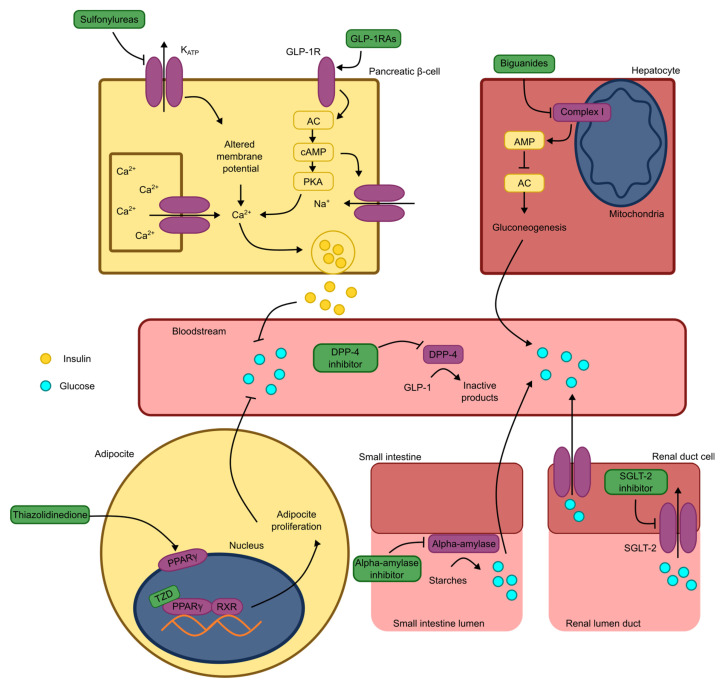
Schematic representation of antidiabetic mechanisms of marketed drugs. Note: KATP: ATP-dependent potassium channel; GLP-1RAs: glucagon-like peptide 1 receptor agonists; GLP-1R: glucagon-like peptide 1 receptor; GLP-1: glucagon-like peptide 1; AC: adenylate cyclase; cAMP: cyclic adenosine monophosphate; AMP: adenosine monophosphate; PKA: protein kinase A; Ca^2+^: calcium ion; Na^+^: sodium ion; DPP-4: dipeptidyl peptidase 4; PPARγ: peroxisome proliferator-activated receptor gamma; TZD: thiazolidinedione; RXR: 9-cis-retinoic acid receptor; SGLT-2: sodium and glucose cotransporter type 2.4.2. Peptides acting through mechanisms involving GLP-1 receptors.

Hepatocytes and muscle cells are particularly important for maintaining blood glucose homeostasis. Hepatocytes serve as glycogen reservoirs capable of supplying glucose to meet immediate metabolic demands [53,54,55]. Biguanides exploit this regulatory capacity by targeting hepatic gluconeogenesis. Their mechanism involves inhibiting NADH–ubiquinone oxidoreductase (Complex 1), leading to increased adenosine monophosphate (AMP) levels and AC inhibition, reducing the cAMP concentrations. The resulting suppression of gluconeogenesis enhances glycemic control in diabetic patients [6]. These mechanisms are fundamental to diabetes treatment and provide valuable insights into the potential mechanisms underlying the action of novel antidiabetic molecules. An illustrated summary of these processes is presented in Figure 2.

Most studies evaluating the effects mediated by GLP-1R have focused on clinical trials involving Exenatide. Among these, studies on individuals with diabetes have demonstrated a significant reduction in their glycated hemoglobin (HbA1c) levels, with decreases ranging from 0.36% to 3.1%. However, other potential benefits associated with GLP-1RAs, such as reductions in body weight, body mass index (BMI), and capillary glycemia, were inconsistent across studies [18,19,20,21]. One hypothesis regarding the limited observation of these additional benefits concerns the relatively short duration of the clinical trials, which typically lasted for around 24 weeks. These effects are often more pronounced with prolonged treatment periods [56,57]. A notable advantage of GLP-1RAs is their dosing regimen, allowing for weekly administration. This convenience is critical in promoting patient adherence to treatment, as fewer interventions reduce the barriers to maintaining consistent therapy [58,59]. Additionally, Exenatide co-administration did not increase the risk of hypoglycemia but was associated with a higher incidence of gastrointestinal side effects.

Taylor et al. (2023) [21] investigated the effects of Exenatide in individuals without diabetes and observed that its administration nearly doubled insulin secretion and accelerated the clearance of glucose from the bloodstream. These effects on the insulin and glucose concentrations followed a biphasic pattern, suggesting that the glucose reduction was directly mediated by insulin action and release. While some drugs enhance the interaction between other substances and specific receptors, Exenatide did not exert such an effect on the insulin sensitivity, as no changes in the insulin responsiveness were detected. The study also revealed considerable inter-individual variability in the drug efficacy. However, this variability could not be linked to homozygous genotypes for the glucagon receptor (GCGR) (p.G40S; rs850763) or the gastric inhibitory polypeptide receptor (GIPR) (p.E354Q; rs1800437) identified in the studied Amish population. These genes are associated with different forms of GLP-1R and GIPR expression and are hypothesized in the literature to contribute to variations in GLP-1RA responses. Nonetheless, this study did not provide sufficient evidence to confirm or refute their involvement. Future research involving larger cohorts of homozygous individuals or studies including heterozygotes is recommended to investigate this hypothesis further.

Gunta et al. (2023) [23] conducted an in silico analysis of four peptides with molecular weights ranging from 6,000 to 8,000 Daltons, derived from snake venom. Their findings indicated that these peptides exhibited less favorable molecular docking interactions with GLP-1R than established GLP-1RAs such as Liraglutide, Semaglutide, Exenatide, and Lixisenatide. However, molecular dynamics simulations involving one of the peptides (P86538) and GLP-1R demonstrated the high conformational stability of the receptor. The study concluded that these peptides are promising candidates for further investigation. Nevertheless, it is suggested that future research should not focus solely on GLP-1R-mediated mechanisms, as the current results do not provide sufficient evidence to confirm their action through this pathway. Additionally, previous studies have reported significant conformational variability in GLP-1R in the presence of well-established agonists [60].

### 4.2. Peptides Acting Through Mechanisms Involving Insulin Receptor Agonism

One of the primary challenges in developing fast-acting insulins is the tendency of insulin molecules to form dimers and hexamers when distributed in the body. For insulin to exert a full and rapid effect, it must remain in its monomeric form [61,62]. In this context, the study of insulin-like peptides (ILPs) derived from animal venoms is particularly relevant, as they may provide insights into developing monomeric insulins or novel therapeutic agents.

Ahorukomeye et al. (2019) [24] investigated seven peptides isolated from the venom of a marine snail (*Conus geographus*). All seven peptides demonstrated the ability to reduce capillary glycemia in a T1DM model using zebrafish. Among these, three peptides—Con-Ins G1, Con-Ins T1A, and Con-Ins K1—induced hypoglycemic effects in a T1DM mouse model. The ability of these peptides to bind to human insulin receptors (hIRs) was also assessed. While activation of hIRs could not be definitively confirmed due to the absence of a critical amino acid sequence in the C-terminal region of the B chain, considered essential for receptor activation, the results were nonetheless encouraging. These findings support further exploration of ILPs for developing monomeric insulins or more stable insulin analogs for biological applications.

Guo et al. (2024) [25] examined the peptide ILP-Ap04, derived from a sea anemone (*Exaiptasia diaphana*). Similarly to Ahorukomeye et al.’s findings [24], this peptide lacked an amino acid sequence deemed essential for receptor activation. Despite this, ILP-Ap04 demonstrated hypoglycemic effects in T1DM zebrafish models. Subsequent in silico molecular docking analyses revealed a strong interaction between ILP-Ap04 and hIRs, characterized by the formation of hydrogen bonds with the receptor target. These two studies complement one another methodologically: while Ahorukomeye et al. (2019) [24] provided evidence for ILPs’ ability to bind and activate hIRs, Guo et al. (2024) [25] elucidated the specific interaction mechanisms through molecular docking analyses. Together, these findings highlight the potential of using venom-derived ILPs as a foundation for developing innovative insulin therapies or enhancing our understanding of insulin receptor interactions.

### 4.3. Peptides Acting Through Mechanisms Involving Potassium Channel Inhibition

Tang et al. (2021) [30] and Ramu et al. (2022) [29] investigated peptides derived from centipedes of the genus *Scolopendra*. The peptides analyzed (SsTx-4, SsTx-4-K14A, SsTx-4- P15A, SsTx-4-Y16A, and SpTx1) were identified as inhibitors of potassium channels (Kir6.2/SUR1), which play a role in maintaining the membrane potential of pancreatic beta cells. These molecules exert their inhibitory effect by binding to the outer vestibule of the channel, thereby blocking its pore. This inhibition leads to changes that typically stimulate insulin secretion [63]. In vivo studies by Ramu et al. (2022) [29] revealed that SpTx1 exhibits a secondary insulin secretagogue function dependent on high glucose concentrations. This finding holds clinical significance, suggesting that this mechanism may reduce the likelihood of severe hypoglycemic events during diabetes treatment. Notably, the in vivo experiments were conducted using a mutant mouse strain with an amino acid alteration in the Kir6.2 subunit (^ENDO^mKir6.2^V108E^) to ensure sensitivity to SpTx1 comparable to that of the human receptor subunit. These findings highlight the extreme specificity of these peptides, as minor modifications in the target protein significantly influence their sensitivity and activity.

The peptide Δ-theraphotoxin-Ac1 demonstrated insulin secretagogue activity in BRIN-BD11 cells through mechanisms involving both K_ATP_ channels and voltage-dependent calcium channels [28]. Pharmacological tools capable of inhibiting adenylyl and guanylyl cyclases revealed a potentiation of Δ-theraphotoxin-Ac1’s effects, suggesting that its action may involve channel regulation and signaling pathways associated with these channels. Insulin secretion mediated by Δ-theraphotoxin-Ac1 was glucose-dependent and effectively regulated capillary glycemia in mice subjected to a high-fat diet and streptozotocin treatment (HFF/STZ), a model resembling T2DM [26,27]. Pharmacokinetic evaluations in healthy mice demonstrated high hepatic, renal, and pancreatic distributions following intraperitoneal (i.p.) administration, as well as distribution across the blood–brain barrier. This latter observation may explain the additional effects of Δ-theraphotoxin-Ac1, such as appetite suppression, potentially mediated by central mechanisms.

Coulter-Parkhill et al. (2023a) [26] studied Jingzhaotoxin IX and Jingzhaotoxin XI using BRIN-BD11 pancreatic beta cells and observed increased insulin secretion mediated by elevated intracellular calcium levels and a prolonged repolarization time in these cells. These findings suggest that these peptides act through interactions with voltage-dependent potassium channels (Kv2.1). Unlike the other peptides targeting potassium channels discussed earlier, Jingzhaotoxins exert their insulinotropic effects independently of the glucose concentrations, raising concerns about their potential to induce severe hypoglycemic events due to their primary secretagogue action [29,30]. Furthermore, determining whether Jingzhaotoxins can promote beta cell growth, proliferation, and protection against apoptosis is particularly relevant given that T2DM progression is associated with beta cell death and reduced insulin secretion [42,43,44]. However, in vivo analyses revealed reduced efficacy in lowering blood glucose levels compared to that in Exenatide-treated controls. These results underscore the challenges of translating in vitro findings into in vivo applications due to significant pharmacokinetic limitations, particularly regarding peptide stability in biological environments [64,65].

### 4.4. Peptides Acting Through Mechanisms Involving Alpha-Amylase Inhibition

Magnificamide has demonstrated the ability to inhibit the enzymatic activity of three alpha-amylases: porcine pancreatic alpha-amylase (PPA), human salivary alpha-amylase (HSA), and human pancreatic alpha-amylase (HPA). It exhibits exceptionally low inhibition constant (Ki) values, indicating its high potency in preventing the catalytic activity of these enzymes, which are responsible for breaking glycosidic bonds in dietary saccharides and starches [31,32]. Notably, magnificamide’s inhibitory potency surpasses that of acarbose, a widely used alpha-amylase inhibitor, with Ki values three to four orders of magnitude lower. However, these findings were obtained in vitro and should be interpreted with caution until validated by in vivo studies [31].

For alpha-amylase inhibitors to exert significant effects on capillary glycemia, they must act within the lumen of the gastrointestinal tract. However, the physicochemical conditions of this environment pose considerable challenges for peptide-based inhibitors [64,65]. Despite these challenges, Sintsova et al. (2023) [32] demonstrated that magnificamide effectively reduced capillary glycemia in a T1DM mouse model. Remarkably, these effects were achieved with significantly lower doses of magnificamide compared to those of standard alpha-amylase inhibitors, highlighting its potential to be a highly effective therapeutic agent.

### 4.5. Peptides Acting Through Diverse Mechanisms

Conlon et al. (2020) [35] evaluated five peptides derived from the venom of *Naja nigricollis*, demonstrating their insulinotropic potential. However, for three of these peptides—Cytotoxin 1, Cytotoxin 2, and Cytotoxin 4—the concentrations required to induce insulin secretion also caused the release of lactate dehydrogenase (LDH) in the tested cell lines, indicating toxicity and potential cell death. Conversely, PLA2-1N and PLA2-2N significantly increased the insulin secretion rates by up to six times the baseline value, without altering the toxicity marker concentrations. Due to limited peptide availability, detailed evaluations of their mechanisms or pharmacological potency were not conducted. Nevertheless, the literature suggests that these phospholipases exert their insulinotropic effects through the lysis of membrane phospholipids (PIP2), releasing arachidonic acid and producing prostaglandin. These products may act via signal transduction pathways involving potassium channels, AC, guanylate cyclase, protein kinases, and other molecular targets [66].

Lugo-Fabres et al. (2021) [34] investigated the peptide s-cal14.2b, derived from the venom of marine snails (*Californiconus californicus*). This peptide induced insulin secretion and regulated the capillary glucose levels during an oral glucose tolerance test in BALB/cAnNHsd mice. Studies on rat pancreatic beta cells revealed that s-cal14.2b likely interacts with metabotropic receptors to regulate calcium conductance in L-type voltage-dependent calcium channels (Cav1.2/1.3) independently of the membrane potential. These findings suggest that s- cal14.2b holds therapeutic potential for treating T2DM in its early stages.

The synthetic peptides HL-7 and HL-10, derived from scorpion venom (*Hemiscorpius lepturus*), were found to induce GLUT4 transporter expression in human skeletal muscle cells (HSkMCs). These peptides also stimulated insulin secretion in INS-1 cells in both glucose-dependent and glucose-independent manners while promoting the expression of mRNA for GLUT4 in soleus muscle cells [33]. The authors concluded that GLUT4 expression correlates with the inhibition of nuclear transcription factor κB (NF-κB), supported by molecular docking analyses showing strong interactions between these peptides and NF-κB. Another proposed mechanism involves metabotropic action on insulin receptors, as Western blot analyses revealed the expression patterns of Akt, mitogen-activated protein kinase (MAPk), and GLUT4 to be consistent with known insulin signaling pathways [40,41].

### 4.6. Limitations, Key Considerations, and Future Perspectives on the Study of Such Substances

**Figure 3:**
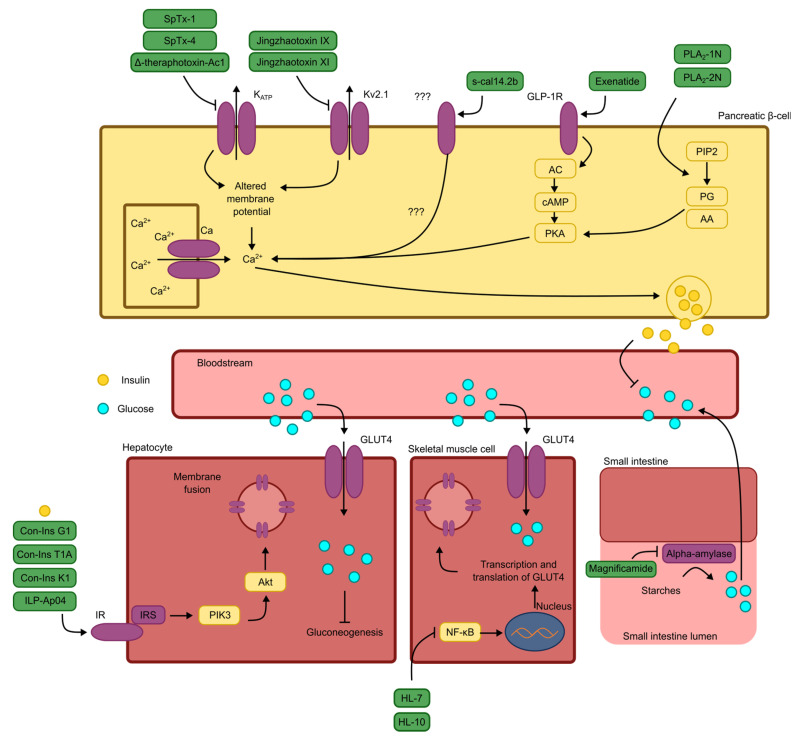
Schematic representation of the mechanisms of antidiabetic action for the peptides discussed, as described by the original authors. Note: KATP: ATP-dependent potassium channel; Kv2.1: type 2.1 voltage-dependent potassium channel; ???: uncertain receptors or signal transduction pathways; GLP-1R: glucagon-like peptide 1 receptor; AC: adenylate cyclase; cAMP: cyclic adenosine monophosphate; PKA: protein kinase A; Ca^2+^: calcium ion; Na^+^: sodium ion; PIP2: membrane phospholipid; PG: prostaglandin; AA: arachidonic acid; GLUT4: glucose transporter type 4; IR: insulin receptor; IRS: insulin receptor substrate; PIK3: phosphatidylinositol 3-kinase; Akt: protein kinase B; NF-κB: nuclear transcription factor κB.

As illustrated in Figure 3, peptides exhibit a wide variety of mechanisms of action, some of which are not aligned with the mechanisms of the currently marketed drugs. This divergence can complicate their detailed exploration, as researchers may lack the appropriate tools or face limited access to necessary resources, thereby hindering the study of these substances [67,68].

There is ongoing concern that substances derived from venoms may exhibit a degree of toxicity similar to that of their source materials. Several of the selected studies assessed the toxicological profile of these peptides using both in vitro methodologies with various cell lines and in vivo approaches in rodent models. For the peptides Δ-theraphotoxin-Ac1, Jingzhaotoxin IX, and Jingzhaotoxin XI, the BRIN-BD11 β-PC line was evaluated for cell viability using MTT and lactate dehydrogenase (LDH) assays across different peptide concentrations. No adverse effects were observed at micromolar concentrations. Additionally, intraperitoneal administration of Δ-theraphotoxin-Ac1 at doses of 2.5, 25, or 250 nmol/kg did not result in any harm to mice [26,27,28]. Similarly, PLA2-1N and PLA2-2N were tested on the BRIN-BD11 β-PC cell line, and no detrimental effects on the cell viability or LDH release were detected at micromolar concentrations [35]. Lugo-Fabres et al. [34] reported that the peptide s-cal14.2b reduced the viability of NIT-1 insulinoma cells, hepatocytes, and β-PCs by approximately 10–20% at concentrations in the µg/mL range. While this reduction was statistically significant compared to the negative control, it remained substantially lower than the effect observed with the positive control, which reduced the cell viability by approximately 90%. The peptides HL-7 and HL-10 were assessed in INS-1 cells and HSkMCs, with no observed loss of viability. Furthermore, no adverse effects were detected in Wistar rats following intraperitoneal administration for seven days at doses in the mg/kg range [33]. Treatment with the alpha-amylase inhibitor peptide magnificamide, administered either intravenously or orally at mg/kg doses, did not produce deleterious effects; the only notable gastrointestinal outcome was an increase in the defecation frequency, which was likely attributable to magnificamide’s impact on the digestion of starches and polysaccharides [32]. Collectively, these findings demonstrate the safety profile of these peptides, supporting their potential for use in diabetes treatment and justifying the continued investigation and development of these bioactive molecules.

It is well established that small molecules generally possess advantages over macromolecules, which include the peptides examined in this study, from both pharmacological and pharmacotechnical perspectives, particularly with respect to their pharmacokinetic properties [69,70]. Nevertheless, despite the challenges associated with translating the use of these macromolecules into clinical research or practice, these peptides exhibit notable advantages, most prominently their high target specificity and substantial pharmacological potency.

The production of peptides is neither straightforward nor cost-effective. Most synthesis processes require expensive reagents and specialized equipment, causing challenges for large-scale production [71,72]. Consequently, studies involving prolonged in vivo treatments, which demand significant quantities of these substances, are scarce. This scarcity negatively impacts scientific progress, particularly in diabetes research. The metabolic and physiological alterations associated with diabetes extend beyond capillary glycemia and include changes in parameters such as glycated hemoglobin, triglycerides, and body weight, as well as disruptions in antioxidant systems and inflammatory responses. Changes in these parameters often require extended periods to manifest and similarly long durations to normalize following treatment initiation [73]. The large-scale production of proteins and peptides has long been regarded as a pivotal objective in modern medicine. In the 1970s, the advent of key biotechnological innovations enabled significant progress in this domain, exemplified most notably by the development of recombinant insulin [74]. Since that time, remarkable advancements have been achieved in recombinant protein technology, leading to increased availability and reduced production costs [75,76]. Whenever feasible, these technological developments should be disseminated and implemented within the scientific community to facilitate sustained, large-scale research endeavors.

Over time, research on peptides has progressed from in silico and in vitro evaluations in earlier studies to in vivo analyses in recent investigations. This progression reflects growing confidence in the potential of these substances for use in diabetes treatment. However, some studies have reported disappointing results. As highlighted by Ramu et al. (2022) [29], a loss of efficacy does not necessarily indicate that these molecules lack therapeutic viability but may instead suggest interactions with different receptor types. Many preliminary in vitro tests use human cell lines with human pharmacological receptors, whereas most in vivo studies involve rodent models, primarily mice, that may not possess identical receptor types. Therefore, discrepancies in the results can be attributed not only to pharmacokinetic challenges but also to pharmacodynamic differences [77]. Research utilizing genetically modified animals engineered to express human-like receptors represents a robust strategy for narrowing the translational gap between preclinical and clinical outcomes, despite the inherent methodological complexity. At present, the majority of these so-called *humanized models* are primarily applied in oncology research [78]. Therefore, provided that financial and ethical requirements are satisfied, such models could be equally well employed in the investigation of novel and effective peptide-based therapeutics for the treatment of diabetes.

Finally, many peptides are derived from animals native to developing countries; however, academic research on these substances is often conducted in wealthier nations with significantly greater resources and funding. This disparity raises ethical concerns about research practices, biopiracy, and neo-colonialism [79,80]. Addressing these issues will ensure equitable and ethical advancement in peptide-based drug discovery and development.

## 5. Conclusions

This investigation presents a comprehensive synthesis of scientific advances achieved over the last five years concerning peptides of animal origin and their antidiabetic mechanisms of action. The analysis determined that these bioactive peptides predominantly exert their therapeutic effects through four distinct pharmacological pathways: (1) glucagon-like peptide-1 (GLP-1) receptor agonism; (2) insulin receptor agonism; (3) inhibition of ATP-sensitive potassium channels (Kir6.2/SUR1) through mechanisms analogous to those of sulfonylurea compounds; and (4) α-amylase enzymatic inhibition. These findings were principally derived from in vitro and in vivo experimental studies, complemented by in silico computational analyses to elucidate the molecular interactions between the target peptides and their respective receptors. The characterized peptides demonstrated exceptional pharmacological potency, exhibiting therapeutic efficacy at remarkably low concentrations, typically within nanogram per kilogram (ng/kg) or nanomole per kilogram (nmol/kg) dosage ranges. Despite the inherent cytotoxic properties associated with animal venoms, the majority of the isolated peptides did not manifest toxicity at the experimentally tested concentrations—a critical parameter for advancing these molecules through the requisite stages of pharmaceutical development. Significantly, the investigation of these peptides need not necessarily culminate in the direct development of novel therapeutics based upon their native chemical structures to yield substantial scientific value. Rather, such studies can provide fundamental insights and conceptual frameworks for designing innovative molecules that exploit specific structural and functional characteristics of these peptides to achieve the desired therapeutic outcomes. Consequently, these venom-derived peptides constitute promising pharmacological candidates with considerable potential for use in diabetes mellitus treatment.

## Figures and Tables

**Table 1:** Key elements in the formulation of the guiding question.

Elements of the Question	Descriptor	Acronym
Peptides derived from animal venoms and their associated mechanisms of action	Problem	P
Diabetic condition	Exposure	E
Clinical, in silico, in vitro, and in vivo studies	Context	Co

**Table 2:** Search strategies used in the Scopus, PubMed, and Web of Science databases.

Search Strategies	Databases
(“venom”) AND (“antidiabetic” OR “diabetes mellitus” OR “diabetes”) AND (“peptide”)	Scopus
(“venom”) AND (“antidiabetic” OR “diabetes mellitus” OR “diabetes”) NOT (Review [Publication Type])	PubMed
(“venom”) AND (“antidiabetic” OR “diabetes mellitus” OR “diabetes”) AND (“peptide”)	Web of Science

**Table 3:** Summary of the data extracted from the selected articles, including information on the peptides studied, doses administered, methodologies employed, observed effects, and probable mechanisms of action underlying the responses.

Article	Sugested Mechanism	OBSERVED EFFECT	Dose	Methodology	Peptide and Animal Source
Joubert et al., 2021 [18].	GLP-1 receptor agonism.	- Reduction in HbA1c, body mass index, weight and post- prandial glycemia; - No difference in hypoglycemic risk or quality of life.	5 μg twice a day for one month and 10 μg twice a day for five months subcutaneously.	Randomised, double-blind, clinical trial.	Exenatide. Gila monster (*Heloderma suspectum*).
Tamborlane et al. 2022 [20].	GLP-1 receptor agonism.	Reduction in HbA1c and insulin dose on treated individuals.	2 mg once a week for 24 weeks (injections without explicit route of administration)	Randomised, parallel-group, phase III study.	Exenatide. Gila monster (*Heloderma suspectum*).
Ekinci et al., 2021 [19].	GLP-1 receptor agonism.	Improved HbA1c management.	2 mg once a week for 20 weeks (injections without explicit route of administration)	Cluster Randomised interventional study.	Exenatide. Gila monster (*Heloderma suspectum*).
Taylor et al., 2023 [21].	GLP-1 receptor agonism.	Increased insulin secretion;	Single subcutaneous administration of 5 μg.	Crossover randomized study with non-diabetic individuals.	Exenatide. Gila monster (*Heloderma suspectum*).
Gunta et al., 2023 [23].	Possible interaction with GLP-1R.	Less favorable interaction between the peptides and the receptor than for the control molecules.	N.A.	Molecular Docking and dynamics in silico with GLP-1R.	Cytotoxin 2a; Cytotoxin 7; Cytotoxin 10. Snakes.
Ahorukomeye et al., 2019 [24].	Direct binding to insulin receptors.	- Reduction in blood glucose in zebrafish; - Less potent interaction between peptides and hIR-B than insulin; - Suggestion of structural characteristics that allow the receptor to be activated after binding with peptides; - A dose 10 times higher than that of insulin reverses hyperglycemia in diabetic mice.	- Single administration of 65 ng/g in zebrafish; - Single administration of 1x, 10x, or 20x the dose of human insulin in mice (1 IU/kg) (no explicit route of administration).	- Zebrafish diabetes model; - In vitro hIR interaction test; - Immunoassay of insulin signaling pathways in NIH 3t3t mouse fibroblasts; - Mice diabetes model (CBA/CaJ and C57BL/6J).	Con-Ins G1; Con-Ins G3; Con-Ins T1A; Con-Ins T1B; Con-Ins T2; Con-Ins K1; Con-Ins K2. Sea snail (*Conus* *geographus*; *C. tulipa*; *C.* *Kinoshitai*).
Guo et al., 2024 [25].	Insulin receptor agonism.	- Significant reduction in blood glucose; - Good interaction with hIR.	Single i.p. administration of 65 ng/g.	- Zebrafish diabetes model; - Molecular docking in silico.	ILP-Ap04. Sea anemone (*Exaiptasia* *diaphana*).
Coulter-Parkhill et al., 2023a [26].	Inhibition of Kv2.1 channels in a glucose-independent manner.	- No impact on cell viability; - Significant increase in insulin secretion in vitro; - Intracellular calcium inflow; - Proliferation, growth and inhibition of apoptosis of β-PCs; - Improved insulin secretion and glucose control in vivo with no effect on appetite.	Single i.p. administration of 25 nmol/kg or 75 nmol/kg in vivo.	- Cytotoxicity and membrane potential assay, assessment of intracellular calcium and evaluation of proliferation and protection against apoptosis in β-PCs (BRIN BD11); - Evaluation of blood glucose concentrations, insulin secretion and appetite suppression in C57BL/6 mice.	Jingzhaotoxin IX; Jingzhaotoxin XI. Chinese tarantula (*Chilobrachys jingzhao*).
Coulter-Parkhill et al., 2023b [27].	Insulinotropic action mediated by K_ATP_ channels.	- Plasma stability up to 12h; - No cytotoxicity with proliferative and anti-apoptotic effects in BRIN- BD11; - In vivo increase in insulin secretion with better glucose tolerance.	Single i.p. administration 25, 75 or 250 nmol/kg	- Peptide plasma stability test; - Cytotoxicity effects	Δ-theraphotoxin-Ac1 (Δ- TRTX-Ac1). Tarantula (*Aphonopelma chalcodes*).
Coulter-Parkhill et al., 2024 [28].	Insulinotropic action mediated by K_ATP_ channels.	- No changes in blood glucose, insulin or glucagon levels; - No changes in insulin sensitivity; - Reduction in body weight and energy demand; - Improved glucose tolerance; - Increased levels of pancreatic insulin and reduced levels of glucagon; - Increase in the size of pancreatic islets and β- PCs.	i.p. administration of 25 nmol/kg twice a day for 28 days.	Glucose tolerance test, insulin sensitivity, insulin and glucagon blood stream levels, and histology and immunohistochemistry in a diabetes model in C57BL/6J mice.	Δ-theraphotoxin-Ac1-OH (Δ-TRTX-Ac1- OH); Δ- theraphotoxin-Ac1- NH 2 (Δ-TRTX-Ac1-NH2) Tarantula (*Aphonopelma chalcodes*).
Ramu et al., 2022 [29].	Inhibition of K_ATP_ channels by ion-conducting pore blocking.	- No effect on blood glucose, insulin secretion or membrane depolarization in pancreatic cells of wild- type mice; - Suppression of potassium currents through the hK_ATP_ channel; - Insulin secretion and membrane depolarization of β-PCs from mice with the V108E mutation; - Reduced glycemia and increased insulin secretion in a mice diabetes model with the V108E mutation.	Single intravenous injection of 1 mg/kg	- Glucose homeostasis in wild mice; - Insulin secretion and electrophysiological assay of β-PCs from wild-type mice; -Electrophysi ological assay of β-PCs from mice expressing human Kir6.2 channel; - Electrophysiological assay and insulin secretion assay in β -PCs from mice with the V108E mutation; -Diabetes model in mice with the mutation V108E.	SpTx1 Centipede (*Scolopendra* *polymorpha*).
Tang et al., 2021 [30].	Inhibition of Kir channels.	Inhibition of Kir6.2/SUR1 channels, Kir1.1 and Kir4.1 in a dose-dependent manner.	N.A.	Electrophysiological tests on HEK293T cells transfected with plasmids for different types of Kir channels.	SsTx-4; SsTx-4-K14A; SsTx-4-P15A; SsTx-4-Y16A. Centipede (*Scolopendra* *subspinipes* *mutilans*).
Sintsova et al. 2019 [31].	Direct inhibition of mammalian alpha-amylases.	Very low inhibition constants for the enzymes evaluated.	N.A.	Inhibition analysis of mammalian alpha-amylases (human salivary alpha-amylase (HSA) and porcine pancreatic alpha-amylase (PPA))	Magnificamide. Sea anemone (*Heteractis* *magnifica*)
Sintsova et al., 2023 [32].	Direct inhibition of mammalian alpha-amylases.	- Potent inhibition of HPA; - Formation of stable complexes between the peptide and the enzymes; - Effective blockade of starch degradation and absence of postprandial hyperglycemia in vivo.	Single oral administration of 0.1, 0.01, 0.005, 0.0025, or 0.001 mg/kg.	- Human pancreatic alpha-amylase (HPA) inhibition assay; - Oral starch tolerance in a mouse model of diabetes (CD-1); - Isothermal calorimetric titration of magnificamide with HSA and HPA.	Magnificamide Sea anemone (*Heteractis* *magnifica*)
Setayesh-Mehr et al., 2023 [33].	Increase in GLUT4 expression by inhibition of NF-κB.	- Glucose uptake by HSkMC; - Significant insulin secretion in INS-1; - Increase in GLUT4, AMPK and Akt expression; - Reduced blood glucose, increased insulin secretion. - In silico interactions with NF-κB.	Daily i.p. injections of 3 or 6 mg/kg for a week	- Insulin secretion test in INS-1 cells; - Glucose uptake in HSkMC - Expression of genes for GLUT4, Akt, and AMPK; - Analysis in a mice model of diabetes; - Molecular docking with nuclear factor κB (NF-κB).	HL-7; HL-10. Scorpion (*Hemiscorpius lepturus*).
Lugo-Fabres et al., 2021 [34].	Possible metabotropic effect	- Reduced cell viability; - Insulin release in NIT-1 cells; - Significant increase in current amplitudes through the Cav1.2/1.3 channels; - Dose-dependent relationship with glucose tolerance - Peripancreatic distribution of administered peptides	Single i.p. injection of 65, 75, 85 or 100 μg per 20 g.	- Cytotoxicityand insulin secretion in NIT-1 cells; - Electrophysiological assay in rat β-PCs; - Glucose homeostasis in BALB/cAnNHsd mice; - Biodistribution in rats.	s-cal14.2b. Sea snail (*Californiconus* *californicus*)
Conlon et al., 2020 [35].	Phospholipa se effects on membrane phospholipids.	PLA2-1N and PLA2-2N stimulate the release of insulin.	N.A.	- Cytotoxicity and insulin release studies in BRIN BD11 cells.	PLA2-1N; PLA2-2N. Spitting cobra (*Naja* *nigricollis*)

Note: N.A.: Not not applicable; HbA1c: Glycated glycated hemoglobin; hIR: hHuman insulin receptor; NIH: National Institute of Health; β-PCs: pPancreatic beta cells; i.p.: intraperitonealIntraperitoneal; GLP-1: gGlucagon-like peptide 1; KATP: ATP- dependent potassium channel; hKATP: hHuman ATP-dependent potassium channel; Kir: iInward rectifier potassium channel; Kv: vVoltage-dependent potassium channel; HEK293T: hHuman embryonic kidney cell line; Cav: vVoltage-dependent calcium channel; HskMCs: hHuman skeletal muscle cells; Akt: pProtein kinase B; GLUT4: gGlucose transporters type 4; AMPK: AMP-activated protein kinase; NF-κB: nNuclear factor κB.

**Table 4:** Analysis using BLAST method of possible homologous sequences in peptides with similar mechanisms of action found in this review.

Article	Possible Homologous Sequence to Other Peptides with Similar Suggested Mechanisms? (BLAST Method)	Peptide Sequence and Molecular Weight	Suggested Mechanism	Peptide and Animal Source
Joubert et al., 2021 [18]; Tamborlane et al. 2022 [20]; Ekinci et al., 2021 [19]; Taylor et al., 2023 [21].	No	MKIILWLCVFGLFLA TLFPISWQMPVESGL SSEDSASSESFASKIK RHGEGTFTSDLSKQ MEEEAVRLFIEWLK NGGPSSGAPPPSG (9478 Da)	GLP-1 receptor agonism.	Exenatide. Gila monster (*Heloderma suspectum*).
Gunta et al., 2023 [23].	No	LQCNKLVPIASKTCPPGKNLCYKMFMVSDLTIPVKRGCIDVCPKNSLLVKYECCNTDRCN (6711 Da); - LKCNKLIPLAYKTCPAGKDLCYKMYMVSNKTVPVKRGCIDVCPKNSLLVKYECCNTDRCN (6792 Da); - LKCNKLVPLFYKTCPAGKDLCYKMYMVATPKVPVKRGCIDVCPKSSLLVKYVCCNTDRCN (6764 Da).	Possible interaction with GLP-1R.	Cytotoxin 2a; Cytotoxin 7; Cytotoxin 10. Snakes.
Ahorukomeye et al., 2019 [24].	Yes (E-value greater than 1e-6 when compared to human insulin)	- MTTSSYFLLMALGLLLYVCQSSFGNQHTRTFDTPKHRCGSEITNSYMDLCYRKRNDAGEKRGRASPLWQRRGSLSKLKARAKRNGAFHLPRDGRGVVEHCCHRPCSNAEFKKYCG (13,134 Da); - MTTSFYFLLVALGLLLYVCQSSFGNQHTRNSDTPKHRCGSELADQYVQLCHGKRNDAGKKRGRASPLWQRQGFLSMLKAKRNEAFFLQRDGRGIVEVCCDNPCTVATLRTFCH (12,816 Da); - MTTSFYFLLMALGLLLYVCQSSFGNQHTRNSDTPKYRCGSEIPNSYIDLCFRKRNDAGKKRGRASPLWQRGGSLSMLKARAKRNEAFHLQRAHRGVVEHCCHRPCSNAEFKKFCG (13,161 Da); - MTTSFYFLLMALGLLLYVCQSSFGNQHTRNSDTPKYRCGSDIPNSYMDLCFRKRNDAGKKRGQASPLWQRGGSLSMLKARAKRNEAFHLQRAHRGVVEHCCYRPCSNAEFKKFCG (13,163 Da); - MTTSSYFLLVALGLLLYVCQSSFGSPHTSDSGTTLVRRRLCGSELVTYLGELCLGNRKRRGFPSMLKARAKRNEAFLLQRDGRGIVEDCCYNDCTDEKLKEYCHTLQG (12,135 Da); - MTTSSYFLLVALGLLLYVCQSSFGNPHTRDSGTTPDRDHSCGGELVDRLVKLCPSNRKRRGFPSMLKARAKRNEAFLLQRDGRVIVGDCCDNYCTDERLKGYCASLLGL (1212 Da).	Direct binding to insulin receptors.	Con-Ins G1; Con-Ins G3; Con-Ins T1A; Con-Ins T1B; Con-Ins T2; Con-Ins K1; Con-Ins K2. Sea snail (*Conus* *geographus*; *C. tulipa*; *C.* *Kinoshitai*).
Guo et al., 2024 [25].	Yes (E-value greater than 1e-6 when compared to human insulin and to Con-Ins G1)	MPRTFLVVLIYILAGFLCSTSALRKVNEASGIKTDGSGYTIVEECCTESCKLEEVNEYCHLFRGRFFCGEQILDIYNTVCNPRSIRRKRSLTVDKREAKKFIRQRR (12,291 Da)	Insulin receptor agonism.	ILP-Ap04. Sea anemone (*Exaiptasia* *diaphana*).
Coulter-Parkhill et al., 2023a [26].	No	ECTKLLGGCTKDSECCPHLGCRKKWPYHCGWDGTF (3960 Da); - ECRKMFGGCSVDSDCCAHLGCKPTLKYCAWDGTF (3733 Da).	Inhibition of Kv2.1 channels in a glucose-independent manner.	Jingzhaotoxin IX; Jingzhaotoxin XI. Chinese tarantula (*Chilobrachys* *jingzhao*).
Coulter-Parkhill et al., 2023b [27]; Coulter-Parkhill et al., 2024 [28].	No	RCLPAGKPCAGVTQKIPCCGKCSRNKCT (2923 Da)	Insulinotropic action mediated by K_ATP_ channels.	Δ-theraphotoxin-Ac1 (Δ- TRTX-Ac1). Tarantula (*Aphonopelma chalcodes*).
Ramu et al., 2022 [29].	Yes (E-value of 2 × 10^−16^ with an identity of 45% with the aminoacids of SsTx-4)	ADLIKKKLPFRTRSKFPRKSECVQDCAKAFTNGNKDKIKDVKSEFFSCYCWYEA (6370 Da)	Inhibition of K_ATP_ channels by ion-conducting pore blocking.	SpTx1 Centipede (*Scolopendra* *polymorpha*).
Tang et al., 2021 [30].	Yes (E-value of 2× 10^−16^ with an identity of 45% with the aminoacids of SpTx1)	EVIKRDIPFKKRKFPYKSECLKACATSFTGGDESRIQEGKPGFFKCTCTFTTG (5983 Da)	Inhibition of Kir channels.	SsTx-4. Centipede (*Scolopendra* *subspinipes* *mutilans*).
Sintsova et al. 2019 [31]; Sintsova et al., 2023 [32].	N.A.	SEGTSCYIYHGVYGICKAKCAEDMKAMAGMGVCEGDLCCYKTPW (4774 Da)	Direct inhibition of mammalian alpha-amylases.	Magnificamide. Sea anemone (*Heteractis* *magnifica*)
Setayesh-Mehr et al., 2023 [33].	N.A.	- YLYELAR (927 Da); - AFPYYGHHLG (1161 Da).	Increase in GLUT4 expression by inhibition of NF-κB.	HL-7; HL-10. Scorpion (*Hemiscorpius lepturus*).
Lugo-Fabres et al., 2021 [34].	N.A.	MNVTVMFLVLLLLTMPLTDGFNIRATNGGELFGPVQRDAGNVLDHGFQRRRECPPRCPTSHCNAGTC (7360 Da)	Possible metabotropic effect	s-cal14.2b. Sea snail (*Californiconus* *californicus*)
Conlon et al., 2020 [35].	N.A.	FADYGCYCGRGGKGTPVDDLDRMGCWPYLTLYKYINANYNINFK (5077 Da); - GGTGTPVDDLDRMGCWPYLTLYKYKCAAAVCNCDLVAANCFAGARYINANAYNINFKKR (6486 Da).	Phospholipa ses effects on membrane phospholipids.	PLA2-1N; PLA2-2N. Spitting cobra (*Naja* *nigricollis*)

Note: N.A.: not applicable; BLAST: Basic Local Alignment Search Tool; GLP-1: glucagon-like peptide 1; KATP: ATP-dependent potassium channel; Kir: inward rectifier potassium channel; Kv: voltage-dependent potassium channel; GLUT4: glucose transporter type 4; NF-κB: nuclear factor κB.

## Data Availability

Original data will be available under request.

## Abbreviations

AA: arachidonic acid; AC: adenylyl cyclase; Akt: protein kinase B; AMP: adenosine monophosphate; AMPK: AMP-activated protein kinase; ATP: adenosine triphosphate; cAMP: cyclic adenosine monophosphate; Cav: voltage-dependent calcium channel; Cav1.2/1.3: L-type voltage-dependent calcium channel; Complex 1: NADH–ubiquinone oxidoreductase enzyme; DPP-4: dipeptidyl peptidase 4; GCGR: glucagon receptor; GIPR: gastric inhibitory peptide receptor; GLP-1: glucagon-like peptide 1; GLP:1R: glucagon-like peptide 1 receptor; GLP-1RAs: glucagon-like peptide 1 receptor agonists; GLUT4: glucose transporter type 4; GMPc: cyclic guanosine monophosphate; HbA1c: glycated hemoglobin; HEK293T: human embryonic kidney cell line; hIR: human insulin receptor; hK_ATP_: human ATP-dependent potassium channel; HPA: human pancreatic alpha-amylase; HSA: human salivary alpha-amylase; HskMCs: human skeletal muscle cells; i.p.: intraperitoneal; ILPs: insulin-like peptides; BMI: body mass index; IR: insulin receptor; IRS: insulin receptor substrate; K_ATP_: ATP-dependent potassium channel; Kir: inward rectifier potassium channel; Kv: voltage-dependent potassium channel; Kv2.1: type 2.1 voltage-dependent potassium channel; LDH: lactate dehydrogenase; MAPk: mitogen-activated protein kinase; Na^+^: sodium ion; NF-κB: nuclear factor κB transcription factor; NIH: National Institute of Health; PECo: Problem (P), Exposure (E), and Context (Co); PG: prostaglandin; PIK3: phosphatidylinositol 3-kinase; PIP2: membrane phospholipid; PKA: protein kinase A; PPA: porcine pancreatic alpha-amylase; PPARγ: peroxisome proliferator-activated receptor gamma; RXR: 9-cis-retinoic acid receptor; SGLT-2: sodium–glucose cotransporter type 2; SUR1: sulfonylurea receptor subunit; T1DM: Type 1 diabetes mellitus; T2DM: Type 2 diabetes mellitus; TZD: thiazolidinedione; β-PCs: pancreatic beta cells.
